# Should we screen for colorectal cancer in people aged 75 and over? A systematic review - collaborative work of the French geriatric oncology society (SOFOG) and the French federation of digestive oncology (FFCD)

**DOI:** 10.1186/s12885-022-10418-5

**Published:** 2023-01-05

**Authors:** Lydia Guittet, Valérie Quipourt, Thomas Aparicio, Elisabeth Carola, Jean-François Seitz, Elena Paillaud, Astrid Lievre, Rabia Boulahssass, Carole Vitellius, Leila Bengrine, Florence Canoui-Poitrine, Sylvain Manfredi

**Affiliations:** 1 Public Health Unit, CHU Caen NormandieNormandie University, UNICAEN, INSERM U1086 ANTICIPE, Caen, France; 2 Geriatrics Department and Coordination Unit in Oncogeriatry in Burgundy, University Hospital of Dijon, Dijon, France; 3 Department of Gastroenterology and Digestive Oncology, Saint Louis Hospital, APHP, Université de Paris, Paris, France; 4 Geriatric Oncology Unit, Groupe Hospitalier Public du Sud de L’Oise, Bd Laennec, 60100 Creil, France; 5 Department of Digestive Oncology & Gastroenterology, CHU Timone, Assistance Publique-Hôpitaux de Marseille (APHM) & Aix-Marseille-Univ, Marseille, France; 6 Geriatric Oncology Unit, Georges Pompidou European Hospital, Paris Cancer Institute CARPEM, inAP-HP, Paris, France; 7 Department of Gastroenterology, INSERM U1242 “Chemistry Oncogenesis Stress Signaling”, University Hospital Pontchaillou, Rennes 1 University, Rennes, FFCD France; 8 Geriatric Coordination Unit for Geriatric Oncology (UCOG), PACA Est CHU de NICE, France; FHU ONCOAGE, Nice, France; 9 Hepato-Gastroenterology Department, Angers University Hospital, Angers, France; 10 HIFIH Laboratory UPRES EA3859, Angers University, SFR 4208, Angers, France; 11 Department of Medical Oncology, Georges-Francois Leclerc Centre, Dijon, France; 12 Public Health Unit, Hôpital Henri Mondor, Assistance Publique-Hôpitaux de Paris, 94000 Créteil, France; 13 Gastroenterology and Digestive Oncology Unit, University Hospital Dijon, INSERM U123-1 University of Bourgogne-Franche-Comté, FFCD (French Federation of Digestive Cancer), Dijon, France

**Keywords:** Harms, Simulation, Cost effectiveness, Acceptability

## Abstract

**Background:**

We have done a systematic literature review about CRC Screening over 75 years old in order to update knowledge and make recommendations.

**Methods:**

PUBMED database was searched in October 2021 for articles published on CRC screening in the elderly, and generated 249 articles. Further searches were made to find articles on the acceptability, efficacy, and harms of screening in this population, together with the state of international guidelines.

**Results:**

Most benefit-risk data on CRC screening in the over 75 s derived from simulation studies. Most guidelines recommend stopping cancer screening at the age of 75. In private health systems, extension of screening up to 80–85 years is, based on the life expectancy and the history of screening. Screening remains effective in populations without comorbidity given their better life-expectancy. Serious adverse events of colonoscopy increase with age and can outweigh the benefit of screening. The great majority of reviews concluded that screening between 75 and 85 years must be decided case by case.

**Conclusion:**

The current literature does not allow Evidence-Based Medicine propositions for mass screening above 75 years old. As some subjects over 75 years may benefit from CRC screening, we discussed ways to introduce CRC screening in France in the 75–80 age group.

**IRB:**

An institutional review board composed of members of the 2 learned societies (SOFOG and FFCD) defined the issues of interest, followed the evolution of the work and reviewed and validated the report.

## Introduction

In Europe, colorectal cancer (CRC) incidence and mortality rates have been stable or have slightly decreased over the past ten years [1, 2]. In France, the same trends are observed with estimated standardized incidence rates for 2018 of 55.3/100,000 for men and 36.7/100,000 for women [1, 3]. The median age at diagnosis was 71 for males and 73 for females, and almost half of CRC occurred after 75 years in women, 40% in men. CRC incidence increase with age: in France from 49.8/100,000 for men [50–54 years] to 414.1 [80-84], and respectively 43.1 to 256.4 for women [4]. Data about adenoma incidence are scarce, a 24-year study in France showed that adenoma incidence increases with age too, and reaches a maximum at 75 years for women and 80 years for men [5]. Given the high incidence rate, the long preclinical phase, the treatable precursor, and a curative treatment for early-stage disease, CRC fulfils the WHO criteria for screening [6] (1: Important health problem, 2: accepted treatment for recognized disease, 3: facilities for diagnosis and treatment, 4: suitable latent and symptomatic stage, 5: suitable test or examination, 6: test acceptable to population, 7: natural history of condition understood, 8: agreed on policy on whom to treat, 9: Cost of finding economically balanced with overall health, 10: case finding should be continuous process).

A comparison with six European countries showed there was no significant difference in CRC net survival. Five-year CRC net survival increased between 1992 and 2004 and reached 64% for colon cancer and 62% for rectal cancer, and the gain was related to a decrease in the excess mortality during the first 18 months after diagnosis for colon cancer and the first 24 months for rectal cancer. This reflects the progress made in the initial management of CRC and underlines the need to implement and improve mass CRC screening [7, 8], which makes it possible to diagnose CRCs at very early stages and to treat them curatively more often with fewer complications.

CRC mass-screening programs have been implemented in several countries worldwide. In 2003, the Council of the European Union recommended that all Member States should establish CRC screening programs for individuals aged 50 to 74 years, with an annual or biennial fecal occult blood test (FOBT), followed by colonoscopy when the results were positive [9]. In 2015, 24 European countries had established CRC screening programs. In the US, opportunistic-based screening programs have been established: participants choose among several options: annual FOBT, multi-target stool DNA test every 3 years, flexible sigmoidoscopy every 5 years, colonoscopy every 10 years, double contrast barium enema or CT colonography every 5 years [10]. In Canada, Chile, Asia and Australia, annual or biennial FIT-based programs have been implemented [11].

In France, an organised national CRC screening programme has been implemented since 2009, for individuals aged 50 to 74 at average risk, using a single-sample FOBT every two years (guaiac FOBT until 2015, FIT since 2015), followed by a colonoscopy if the result is positive. The chosen cut-off value is 30 µg Hb/g feces with the OC-Sensor®.

The CRC mass screening participation rate in France is low, 34.6% for the 2020–2021 round, far from the 45% rate recommended by the European Council as the minimum target rate. This rate is better for females (35.7%) than for males (33.5%) and increases with age from 31.9% [50–54 years] to 39.6% [70–74 years] for males and 33.5% to 39.5% for females. (https://www.santepubliquefrance.fr). The proportion of subjects fulfilling exclusion criteria for CRC screening increased with age, from 5.4% [50–54 years] to 25.1% [70–74 years] for males and from 6.3% to 21.3% for females.

The positive predictive value of a positive test for CRC increased with age, from 4.0% [50–54 years] to 10.2% [70–74 years], and from 27.6% [50–54 years] to 31.9% [70–74 years] for advanced adenoma (≥ 1 cm, high-risk dysplasia, or villous component).

The French population is ageing and life expectancy at 75 years is increasing, reaching 12 years for men and 14.8 years for women, and respectively 8.9 years and 10.1 years at 80 years (https://www.insee.fr/fr/statistiques accessed in September 2020). The increased incidence of CRC and advanced adenoma with age suggests that screening people over 75 years would be beneficial, while the decrease in residual life expectancy suggests the opposite.

**Fig. 1:**
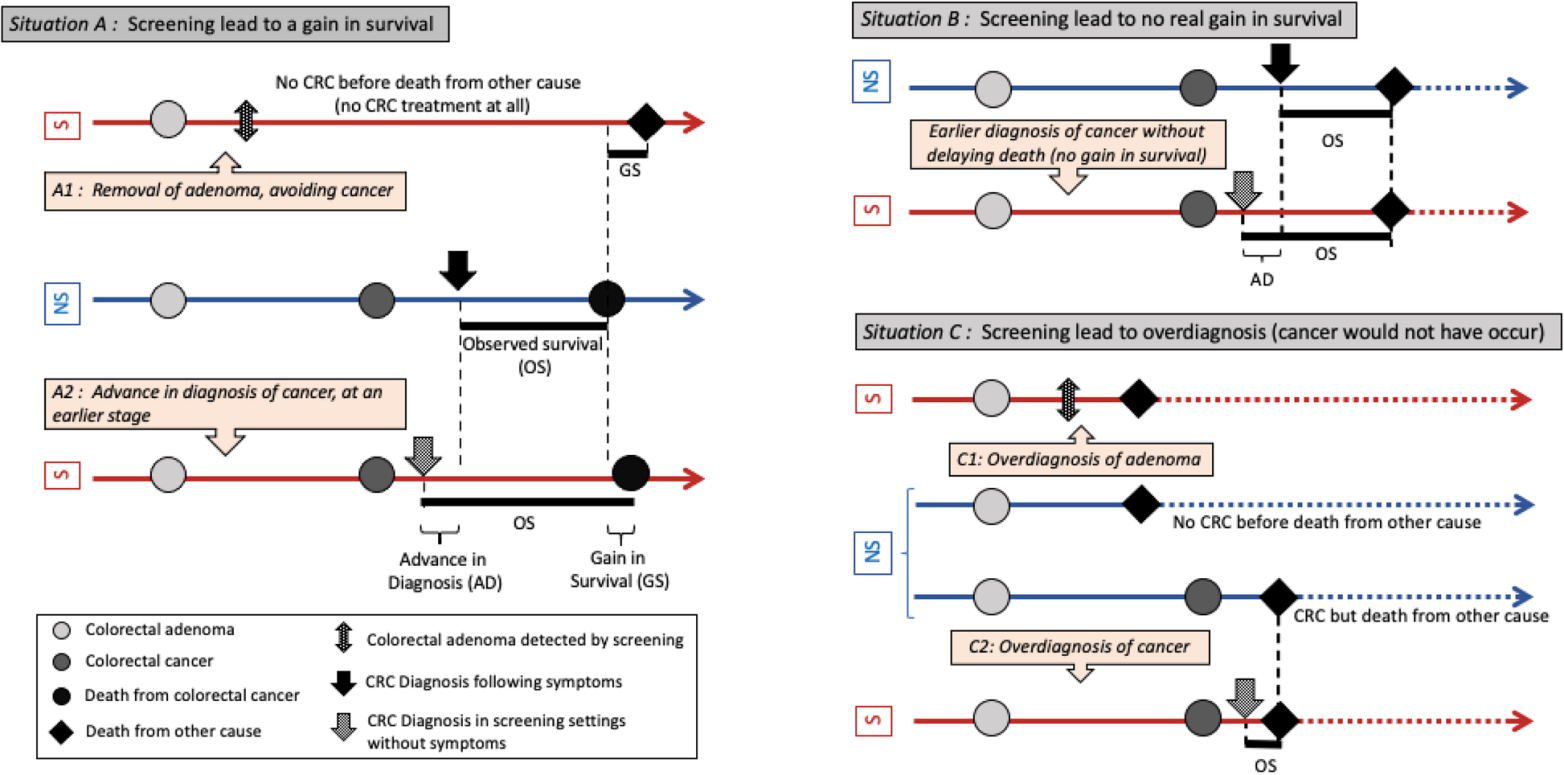
Screening and natural history of colorectal adenoma and cancer

Harm associated with screening includes fear and anxiety, inconvenience, and complications due to the diagnostic procedures. A more complex risk of screening is overdiagnosis and overtreatment (Fig. 1). Overdiagnosis is defined as a diagnosis of a disease which would not have been detected in the absence of screening during the whole life of the subjects, and would have no consequence on his life expectancy [12, 13]. Overdiagnosis may induce overtreatment, as defined by treatments applied to patients in the context of overdiagnosis, which have no impact on their health/life. Overdiagnosis and overtreatment are statistical concepts, and it is not possible to state, for a specific subject, whether the detected lesion would have been detected in the absence of screening or not. Considering the life expectancy of elderly, the risk of overdiagnosis is certainly greater.

The aim of our work was to update knowledge about CRC screening in older individuals by conducting a review of the literature and to make recommendations.

## Methods

The benefit-risk ratio of CRC screening in the elderly was based on a search of the literature concerning key principles in cancer screening: detection of cancer in asymptomatic individuals, analysis of the benefit-risk ratio. CRC screening strategies include one-step screening by colonoscopy, or two-step screening with a first triage test followed, when positive, by a colonoscopy.

The literature search was conducted using the PUBMED database, to identify review articles published on colorectal cancer screening in the elderly. The methodological filters used were English, Review and Meta-analysis. The relevant articles were extracted in October 2021. The search equation combined Medical (MeSH) terms as follows: ("Aged"[Mesh] OR "Aged, 80 and over"[Mesh] OR "Geriatrics"[Mesh] OR elderly) AND (“Mass screening”[Mesh] OR screen*) AND ("Colorectal Neoplasms/diagnosis"[Mesh] OR “colorectal cancer”).

The titles and abstracts of the 249 articles retrieved were analyzed by three physicians (VQ, LG, SM), to select relevant articles for a population older than 75 years old. Cross-references and related articles were used to enrich the literature search, and complementary searches were performed to find other articles on the acceptability, efficacy, cost-effectiveness, and harms of screening in this population, together with the state of international guidelines. The questions addressed by the working group were: What observed data are available? What are the side effects of screening in this population? How effective is screening in this population? What is the cost-effectiveness of screening in this population? What acceptability? What are the results of existing reviews? What are the international recommendations?

## Results

### Observed data on CRC screening in the elderly

There is no randomized controlled trial on the efficacy of extending CRC screening over 75 years. Of the four RCTs on efficacy of FOBT screening, only the Minnesota trial [14] evaluated screening until 80 years, and no data are available on the specific results in the 75–80 years age subgroup. The few data available on CRC screening in elderly subjects [15–17] demonstrated larger detection rates of significant adenomas and CRC in elderly than in the younger population (about 5% to 10% increase) (Table 1).

**Table 1:** Observed data on colorectal cancer (CRC) screening in the elderly

Study	Type of study	Population	FOBT positivity rate / colonoscopy rate	Lesion detection rate
Lin, 2006 [16]	Cross-sectional monocenter study	1,244 average-risk asymptomatic colonoscopy-screened subjects (147 aged 75–79, and 63 over 80y,) in one US center	NA	Advanced neoplasia (including CRC) rate: 3.2% in 50-54y, 4.7% in 75–79 and 14.3% in 80 + 2 invasive cancers in 50–54 and 1 in 80 +
Kirstler, 2011 [17]	Longitudinal cohort study (7 years of follow-up)	212 average-risk subjects (75 aged 70-74y, 93 aged 75-79y, and 44 aged over 80y) with positive FOBT in four US centers	Colonoscopy rate after positive FOBT: 56%	Significantadenomas rate: 29% in 70 + (of which 79% still alive at 5 yrs) 6 cancers 46% of patients without colonoscopy died from other cause than CRC within 5yrs after the test
Koivogui, 2019 [15]	No follow-up data	18,704 average-risk subjects corresponding to 18,995 FOBT tests in 11 French districts (15,855 aged 75y, 2,545 aged 76-80y, and 595 aged 80y)	FOBT positivity rate: 3.7% in this 75 + , vs 2.9 overall Colonoscopy rate: 81.3% in 75 + , vs 81.9% overall	Neoplasia rate: 19.3% in 75 + , vs 14.1% overall Cancer rate: 3.7% in 75 + , vs 1.8% overall Predictive positive value (PPV) for CRC: 12.2% in 75 + , vs 7.4% overall PPV for neoplasia: 63.7% in 75 + , vs 58.8% overall

*CRC* colorectal cancer, *FOBT* fecal occult blood test, *PPV* predictive positive value

### Adverse events associated with endoscopic procedures in the elderly population

Serious adverse events comprise perforation, bleeding, cardio-vascular event, pulmonary event, general anaesthesia-related event, and death (Table 2). These adverse events can outweigh the benefits of screening. Adverse events rates reported in the elderly frequently came from studies in which the colonoscopy was done for reasons other than screening alone. The great majority of guidelines, reviews and studies report an increased risk of serious endoscopic-related complications after 75 years [18–36]. The risks of perforation and bleeding doubled after 75 years (10.3/10,000) compared to 70–74 years (5.6/10,000) [27,33,34]. Adverse events from colonoscopy increase by 10% after age 65, and the risk of perforation by 30% [26,31]. Cardiovascular and pulmonary complications related to anaesthesia increased from 26/1000 after 65 years to 35/1000 after 80 years [26,31].

**Table 2:** Risk of colonoscopy-related complications for 1 000 individuals

Study	Type of study	Population	Perforation (‰)	Gastrointestinal bleeding (‰)	Cardiovascular (CV), respiratory (R) (‰)	Death (‰)
Day LW, 2011 [25]	Review All indications		≥ 65y: 0 to 6.6 ≥ 80y: 0 to10.5	≥ 65y: 0 to 14.9 ≥ 80y: 0 to 9.1	≥ 65y: 25.9 ≥ 80y: 34.8	≥ 65y: 0 to 1 ≥ 80y: 0 to 9.7
Ure T, 1995 [35]	Case–control All indications	354 ≥ 70y 302 < 70y	0 0	0.2 0.2		0 0
Warren JL, 2009 [31]	Population-based All indications 66–95 years	53,220	0.1	1.8	CV: 16.6	
		Screening population (*n* = 5,349)	2.8 66-69y: 5.0 70-74y: 5.8 75-79y: 7.2 80-84y: 8.8 ≥ 85y: 12.1		CV: 12.5 66-69y: 12.6 70-74y: 16.0 75-79y: 20.6 80-84y: 25.7 ≥ 85y: 31.8	
Rutter CM, 2012 [23]	Cohort Screening and follow-up colonoscopy 40–85 years	43,456 (158,295 colonoscopies)	0.5 40-49y: 0 50-64y: 0.3 65-74y: 1.0 75-85y: 1.7	2.8 40-49y: 2.3 50-64y: 2.1 65-74y: 4.3 75-85y: 8.1		0.3 40-49y: 0 50-64y: 0.3 65-74y: 0.4 75-85y: 1.3
Ko CW, 2010 [24]	Cohort Screening and Follow-up colonoscopy > 40 years	21,375	0.19	0.6	CV: 4.9	
			40-59y: 1.2 60-69y: 1.8 70-79y: 3.5 ≥ 80y: 4.4		R: 7.5	
Garcia-Albeniz X, 2017 [32, 33]	Population-based screening population 70–79 years	1,355,692	70–74: 0.4 75–79: 0.4	70–74: 0.4 75–79: 0.5	70–74: 10.7 75–79: 18.1	
Causada-Calo N, 2020 [34]	Population-based cohort Individuals with average colorectal cancer risk	30 443: 50-74y 7 626 ≥ 75	0.4 0.8 NS	3.0 9.0 *p* < 0.001	CV: 5.0 CV: 18.0 *p* < 0.001	

For two authors, age alone must not be considered as a factor that increases the risk of colonoscopy adverse events [23, 37, 38]. For others, age is an independent factor associated with an increase in colonoscopy-related perforation and bleeding [23, 39]. For the authors of the American Cancer Society guidelines 2018, age and comorbidity increase the risk of colonoscopy adverse events and must be considered together and not separately [33]. In addition Day LW et al. mentioned that colon cleansing is more difficult to achieve in the elderly, considered insufficient in 4% to 57% of studies, and that a complete colonoscopy is less frequently achieved, in only 78% to 86% of cases [26, 29].

The use of computed tomographic colonography (CTC) is proposed to reduce the risk of optical colonoscopy (OC), especially the risk of gastrointestinal bleeding, perforation and cardiovascular events [40, 41]. A meta-analysis found a 0.5% risk of severe adverse effects in individuals over 65 years, 0.2% in asymptomatic individuals [42]. Two large non-randomized studies found no significant difference in perforation rates between CTC and OC. This may be due to better detection of micro-perforation with no clinical consequences [40, 41]. CTC requires neither intravenous analgesia nor sedation, but spasmolytic agents may be used to improve image quality [41]. Spasmolytic agents have anticholinergic effects, with potential cardiac and ocular side-effects, as well as antimuscarinic effects on the urinary bladder. Bowel preparation is similar to that for optical colonoscopy, and in case of neoplasia detection, an optical colonoscopy is required to further explore the lesion, or to perform polypectomy. Furthermore, CTC can lead to the detection of extracolonic lesions.

### Effectiveness of screening in this population

The only available data on the benefit-risk of CRC screening in this population arise from simulation studies [17, 18, 24, 43–47] (Table 3). In these studies, the sensitivity and specificity of tests were assumed not to vary with age, although a decrease in specificity might be observed in the elderly for FITs, for example, due to other causes of bleeding [23]. All of these studies excepted one [44], supposed adherence to the screening strategy from the age 50 until the stipulated age for the end of screening. Data used in simulations were US data only for both CRC incidence/mortality, and life expectancy.

**Table 3:** Simulation results on colorectal cancer (CRC) efficacy, effectiveness and cost-effectiveness in the elderly

Study	Screening test	Simulation methods, and underlying assumptions	Efficacy based on simulation results
Ko, 2005 [23]	FOBT, flexible sigmoidoscopy, colonoscopy	US life expectancy tables, SEER program CRC mortality rates Decrease in CRC mortality at 5 years (assumption): - FOBT 18% - Flexible sigmoidoscopy 40% - Colonoscopy 75% No decrease in mortality in the first 5 years after the screening test	Number needed to screen increased with age and with the reduction of life expectancy Colonoscopy-related complications increased with age Complications outweighed benefits for - Women 75-79y, men 70-74y in poor health status - Women 85-89y, men 90-94y in average health status No benefit from screening if life expectancy < 5 years
Lin, 2006 [16]	Colonoscopy	US life expectancy tables / SEER program CRC mortality rates Life expectancy estimated with and without screening according to sex, age and colonoscopy findings, considering polyp lag time and transforming rate, No complication of colonoscopy was simulated	Simulated life expectancy extension: - 0.85 years (2.9%) in 50-54y - 0.17 (1.6%) in 75-79y - 0.13 (1.7%) in ≥ 80y Patient benefit: - 6.0% in 50-54y - 12.2% in 75-79y - 15.9% in ≥ 80y Number of colonoscopies per life year saved - 1.18 in 50-54y - 5.77 in 75-79y - 7.95 in ≥ 80y
Zauber, 2008 [42]	FOBT, flexible sigmoidoscopy or colonoscopy	US life expectancy tables / SEER program CRC mortality rates, MISCAN and SimCRC models, Sensitivity and specificity supposed constant according to age	Increase in number of colonoscopies (D-COL) and in LYG (D-LYG) by extending CRC screening from 50–75 to 50-85y age / 1.000 subjects Biennial FIT: - D-COL = 212 D-LYG = 8 (+ 4.0%) - Efficiency ratio D-COL/D-LYG = 26,5 Decennial colonoscopy: - D-COL = 398 D-LYG = 6 (+ 2.6%) - D-COL/D-LYG = 66.3 No efficient 50–85-y screening program compared to 50-75y
Kirstler, 2011 [17]	FOBT	Cohort observed data (no simulations) of FOBT positive individuals with 7 years of follow-up Benefit: observed survival of at least 5 years after the diagnosis of a colorectal neoplasia (advanced adenoma or CRC)	Mean benefit of screening for 15.6% of individuals: - 10% for those with the worst Charlson score - 20% for those with the best score
Lee, 2013 [46]	FOBT	Meta-analysis of survival data observed in CRC screening randomized controlled trials Time Lag to benefit based on MCMC models Life expectancy simulators (eprognosis.com) [47]	Time lags to benefit of absolute risk reduction: - 4.8 years (2.0 to 9.7) needed to prevent one colorectal cancer death for 5000 people screened - 10.3 years (6.0 to 16.4) needed to prevent one colorectal cancer death for 1000 persons screened
Van Hees, 2014 [43]	FOBT, flexible sigmoidoscopy or colonoscopy in unscreened elderly	Autopsy studies, CRC survival data from SEER program [48] US comorbidities status specific life tables established in 2013 from Medicare population [49]. MISCAN-Colon model calibrated with incidence CRC data observed in the 1975–1979 US SEER program (before introduction of screening), Threshold of willingness to pay: $100,000 /QALY gained	LYG for once-only screening per 1,000 subjects at the age of 80 in subjects with no comorbidity: - Colonoscopy: 52.9 LYG - FIT: 22.5 LYG QALY for once-only FIT screening per 1,000 subjects at the age of 80 according to comorbidity: - Colonoscopy: 46.9 (no comorbidity) to 13.9 (severe comorbidity) - FIT: 19.2 (no comorbidity) to 6.7 (severe comorbidity) Cost-effectiveness cutoff: - No comorbidity 86y - Moderate comorbidity 83y - Severe comorbidity 80y
Van Hees, 2015 [44]	Colonoscopy	SEER program CRC incidence data, MISCAN-Colon model Threshold of willingness to pay: $100,000 /QALY gained	Benefit of screening decreased with age, and harms were greater than benefit for ages between 80 and 85y Screening was cost effective - for individuals without prior screening compared with individuals with prior screening - for individuals without comorbidities compared with individuals with comorbidities
Knudsen, 2016 [45]	Colonoscopy and CT colonography, FOBT, Sigmoidoscopy,	CRC data in US SEER program, US life table. SIM-CRC, MISCAN-Colon and CRC-SPIN models	For persons adequately screened up to age 75 years, additional screening yielded small increases in LYG relative to the increase in colonoscopy burden This suggests that 75 years would be a reasonable age to end screening
Cenin, 2020 [50]	Biennal FIT	MISCAN-Colon	Optimal stopping age for colorectal cancer screening: - 66 years for unhealthy individuals with perfect screening history - 90 years for healthy individuals without prior screening

*CRC* colorectal cancer, *FIT* Fecal immunochemical test, *FOBT* Fecal occult blood test, *LYG* life year gain, *MCMC* Markov Chain Monte Carlo, *QALY* Quality adjusted life year

The benefits of screening decreased with age and comorbidity. The potential for screening-related complications was greater than the estimated benefit in some population subgroups aged 70 years and older. Nonetheless, at all ages and life expectancies, the potential reduction in mortality from screening outweighed the risk of colonoscopy-related death. Colonoscopy screening provided the greatest benefit but also the highest risk of complications.

One of these simulation studies determined up to what age CRC screening should be considered in unscreened elderly people according to comorbidities and the choice of test [44]. The effectiveness and the cost-effectiveness of the screening program decreased with age, and colonoscopy was the most effective and most expensive. The decline in effectiveness is explained by the increased risk of death from other causes with age, the harms of colonoscopy, overdiagnosis and overtreatment. Screening remains effective in populations without comorbidity. Van Hees et al. also showed that comorbidity was an important determinant of the harms and benefits of screening in the decision to stop screening [44].

A recent U.S. simulation model that included screening history and life expectancy revealed a wide age range for stopping screening, between 66 and 90 years of age. The subjects ranged from unhealthy people with perfect screening histories to healthy people with no prior screening [47].

Finally, one simulation study evaluated the age at which screening provided similar harms and benefits to those found with screening at age 74 in average-health individuals. The findings indicated that screening should be stopped before 75 in some populations, and even before 70 years in subjects with severe comorbidities [52].

### Cost-effectiveness

Taking a willingness-to-pay threshold of $100.000/QALY, it has been shown that screening with colonoscopy remains cost-effective with increasing age, even in the oldest age groups [53]. In such cost-effectiveness analyses, the impact of the reduced life expectancy in the elderly is compensated by the higher diagnostic yield of screening endoscopy. However, screening colonoscopy in elderly persons (aged > 80 years) results in only 15% of the expected gain in life expectancy reached in younger patients [17].

Two reviews concluded that screening in subjects over 70 years [23] or 75 years [54] was not or less cost-effective than in younger people, with a low level of evidence. The 2019 simulation-model cost-effectiveness study done in France concluded that extending the upper-age limit to 80 years in a FIT/2 years-based screening program, with a 30% participation rate would result in a 5% decrease in CRC mortality, a 1% decrease in CRC incidence, and a 16% increase in cost [55].

### Acceptability of screening

A cohort study [56] of 27,404 individuals aged 65 years or more, screened for breast, prostate, cervical or colorectal cancers showed that CRC screening was continued in 50.8% to 40.8% according to their 9-year mortality risk, and in 48.5% to 40.8% according to their 5-year mortality risk. The only factor associated with the discontinuation of screening was age greater than 80 years for both 9- and 5-year mortality risk. Similar findings were observed in another study on 27,068 Veterans older than 70 years of age in the USA [57].

In a French study, 4,268 individuals aged 70–74 years with no prior screening were invited in 1988 to do a Hemoccult® test every 2 years until 2002 (they were aged 84–88 years at this time), and were followed up until 2009. The participation rates were good (≥ 40%) up to age 78 and satisfactory (≥ 30%) up to age 80. A modest (10%) not significant reduction in mortality was observed (standardized mortality ratio 0.90 [95%CI 0.73–1.11]). No data about comorbidities were available [58].

The acceptability of repeated colorectal cancer screening with a FOBT, in a population aged 60 to 70 years could be based on perceptions of remaining life expectancy. Those expecting to reach ≥ 90 were more likely to accept repeated screening than those expecting to die before 80 years [59]. In elderly patients, information affected perception of the efficacy of screening tests but not their preference [60].

Data on colonoscopy rate after a positive FOBT are scare, and divergent (see Table 1) [16, 18].

### Synthesis of data from narrative reviews

Some reviews [22, 61, 62] concluded that there were no sufficient data in the literature to recommend screening after 74 years. Other reviews concluded that there was probably a screening benefit for the elderly especially if they had no history of screening and if their life expectancy was at least 10 years [23, 27, 29, 63, 64], or at least 7 year [19]. The benefits of screening decrease with age, significantly after 80 years, and become null at 85 years for men and 90 years for women [27, 29]. Pasetto et al. [65] reasoned that screening is efficient until 80 years for individuals not up to date with screening, with a 5-year life expectancy and fit enough to undergo a potential curative treatment for CRC.

Day et al. reported that 80% of the expected screening benefit is reached when screening ends at 80 years [27, 29]. The great majority of reviews concluded that screening between 75 and 85 years must be decided case by case, considering age, comorbidities and life expectancy [19, 23, 27, 29, 63].

### Synthesis of international guidelines

An analysis of international guidelines [66] and ongoing CRC screening programs [11] revealed differences between countries with free-of-charge organized screening programs ending at 75 years old or even earlier, and countries with an opportunistic insurance/copayment screening system based on individual decision-making and extending to as late as 85 years (Table 4).

**Table 4:** Comparison of upper age limit in ongoing screening programs or guidelines according to countries

	** ≤ 70 years**	** ≤ 74–75 years**	** ≤ 80 years**	** ≤ 85 years**	**No upper age limit**
Europa [67, 68]	*Finland*, *Georgia*, Greece, *Hungary*, *Italy*, *Kazakhstan*, Malta, *Norway*, *Poland*, *Portugal*, *Slovenia*, *Spain*, *Sweden*, *Turkey*	*Belgium*, *Croatia*, Denmark, *Estonia*, *France*, Iceland, *Ireland*, Israel, Lithuania, Luxembourg, *Netherlands*, *Russian Federation*, *Serbia*, *United Kingdom* European Council guidelines [9]	Monaco, San Marino, Switzerland		Austria, Bosnia and Herzegovina, *Cyprus*, Czech Republic, Germany, Latvia, Republika Sprska, Slovakia Republic
America [11]		USA—ACP guidelines [69]: CRC not recommended as soon as the life expectancy is estimated less than 10 yearsUSA—ACP guidelines [51]: CRC screening not recommended as soon as estimated life expectancy is less than 10 years Canada—CTFPHC guidelines [70]: CRC screening after 75 years is not recommended *but can be discussed*		USA—ACS, USPSTF and NCCN guidelines [28, 71, 72]: *Screening in the 76–85 years age group is an individual decision* USA—MSTFCC guidelines [73]: Screening in the 76–85 years age group considered in individuals without prior screening	
Asia [11, 74]	Hong-Kong, Saudi Arabia [20]	Australia, China, Taiwan			Japan, South Korea, Thailand

Countries in which CRC screening is organized, rather than spontaneous-opportunistic are written in italic

Payment policy: ^*^free of charge, versus ^$^insurance/copayment

In South Korea, on organized free of charge screening program is proposed to most deprived, while others may perform spontaneous screening^97^

The European Council recommended screening until the age of 75 [9], and this cut-off is observed in most European countries [67, 68], except for countries that opted for individual-choice screening, which in most cases is not free of charge. In Asia, there tend to be organized screening programs to the age of 75 or 70 years, especially in deprived populations. These often coexist with screening based on individual choice with no upper age limit [74]. In Canadian guidelines, screening after 75 is not recommended but can be discussed [70]. In the USA, CRC screening in the 76–85 age group: 1/ should be an individual decision and can be discussed, according to the NCCN (National Comprehensive Cancer Network), ACS (American Cancer Society) and USPSTF (U.S. Preventive Services Task Force) guidelines, 2/ should be considered for individuals without prior screening, according to the MSTFCC (Multi-Society Task Force on Colorectal Cancer), but is not recommended, according to the ACP (American College of Physicians) guidelines.

Because of the reduction in the expected benefits and the increased risk of colonoscopy-related complications with age [19, 29, 63], most guidelines on screening in subjects aged 75-85y recommend an individual decision based on life expectancy of at least 10 years [20, 21, 28, 70, 73], comorbidities [28, 73] and the history of screening [21, 28, 70, 73].

## Discussion

The lack of data on CRC screening in the elderly needs to be addressed. In studies considering patients more than 75 years old, most are just above 75, and the proportion of subjects older than 80, is low. Most of the data come from simulations. Furthermore, the scarce available data focus on colonoscopy screening in private healthcare systems that concentrate on individual choice, as in the United States. Most guidelines, especially in countries with a single-payer healthcare system and organized screening, define a fixed-age cut-off for cancer screening at 75 years. In private healthcare systems, extending CRC screening until 80 or even 85y is proposed, with the decision being left to the individual.

The population above 75 years is characterized by its heterogeneity in terms of comorbidities and frailties [75]. It was estimated that a third of the subjects 75 + in the USA have a life expectancy of more than 10 years [76]. The estimated life-expectancy at age 75 years decreases with the number of comorbidities [50, 77]. Multiple chronic conditions not only reduced life expectancy but often increased Disabled Life Expectancy [78].

In France, the organized screening program relies on systematic invitations until the age of 75 years. No specific information is systematically given in the final invitation to inform participants that it is the last invitation. After 75 years the call-recall process simply ends and the centralized laboratory does not analyze screening FITs after 75 years. However, further CRC screening demand occurs after the age of 75 years, from either former regular participants of the organized screening program, or non-participants.. Subjects at high-risk of CRC or with a history of adenoma are excluded from organized screening as they undergo specific regular colonoscopy screening/surveillance. If the last colonoscopy at the age of 75 is normal, this surveillance stops. However, if an adenoma is detected, the follow-up continues and, as in other countries [79], French recommendations do not specify an upper age limit, but stipulate the need to justify the continuation of endoscopic follow-up, taking into account the benefit/risk ratio and, in those aged 80 years or more, a life expectancy of at least 5 years is required [80].

As some subjects over 75 years may benefit from CRC screening, we discussed ways of introducing CRC screening in countries using a FIT-based screening program in the 75–80 age group, based on the following key principles:

- increasing participation in the 50–74 years old population is the priority (the French participation rate is only 30%), including for the last invitation at the age 74,
- continuing screening after 75y should not be systematic,
- decisions to propose screening after 75 years should systematically involve the general practitioner (GP) in charge of the patient, personally, even for former regular participants,
- one-time colonoscopy to end screening does not seem compatible with the French CRC screening program, for several reasons including acceptability, risk and costs.

The organized CRC program could be adapted and continued in the 75-80y age group. However, there is no simple, validated way to identify in the health insurance database individuals unlikely to benefit from screening. Therefore, instead of inviting subjects directly, the invitation could be sent to GPs, who would then select among their patients those who meet screening criteria. Alternatively, CRC screening after 75y could be left to the discretion of the GP, without involving a centralized structure.

The first challenge is to guide the physician in selecting subjects who could benefit from screening, keeping in mind the necessary clinical eligibility of the patient for colonoscopy (including anesthesia) in case of a positive FOBT, together with the risk of overdiagnosis and colonoscopy complications. First, subjects with geriatric syndromes should be excluded from screening. Then, frailty detection tools can be used by GPs, such as the timed up and go test (TUG) or the Gait Speed Test (GST) [81, 82], simpler than the time-consuming Comprehensive Geriatric Assessment (CGA). The Activities of Day Living (ADL) [83] and Instrumental Activities of Day Living (IADL 4 items) [84] scales are also useful, as functional status correlates with overall survival and complications of oncologic treatments [85]. Finally, user-friendly models can estimate life expectancy, expected to be greater than 10y to propose screening.

Among the prognostic indices for community-dwelling older adults identified by the review of Yourman [86], only two can predict outcomes over the coming 9 to 10 years [87, 88]. As these models were built in the United States, they may not be suitable for the French population.

Once elderly people who would benefit from CRC screening have been identified, physicians must adequately communicate with them to explain the choices available. The ability to discuss stopping cancer screening, even in patients younger than 75 years, is an important issue for clinicians [89]. When proposing screening, physicians should raise the issue of the patient’s expectations. Even in regular participants (the proportion of whom increases with age [90]), cancer screening after 75 years should not turn into an automatic “routine”. The benefit-risk ratio evolves with age. For example, the elderly would be unlikely to benefit from prophylactic removal of adenomas, as this intervention would have a limited impact on CRC incidence and life expectancy [17], whereas the risk associated with colonoscopy increases. Also, the value individuals place on prolonging life may vary [91]. Some subjects may favor relatively good health in the short term, compared with a prolonged but poorer quality life due to treatment. Information tools should be developed and tested in this population, in order to foster informed decision-making [92].

Should CRC screening be extended to 75 years, the impact on the healthcare system would deserve further attention, and perhaps other health concerns should be prioritized in this specific population. The costs of FIT and their impact on the health care system are far less a matter of concern than is the impact of the colonoscopies induced by this strategy. The availability of colonoscopy resources and the overall costs of these diagnostic procedures need to be verified and would depend on the participation rates in this population. In addition, individuals who regularly participate in CRC screening are those who invest in their health and who would probably have a better life expectancy. The existence of a social gradient in cancer screening participation, and in life expectancy has been well demonstrated [90, 93].

## Conclusion

Considering the current literature, there is no Evidence Based Medicine to justify CRC screening beyond 75 years old. The hypotheses established in simulation studies deserve to be verified in prospective studies. There is probably a place for organized mass-screening strategies, with additional exclusion criteria, and/or for opportunistic screening for individuals aged 75–80 years, considering life expectancy and screening history. Screening studies dedicated to the elderly are needed in order to define easy tools and decisional algorithms to underpin practitioners’ decisions.

## Data Availability

On request from Lydia Guittet and Sylvain Manfredi.
